# Synthesis and Characterization of Silver and Graphene Nanocomposites and Their Antimicrobial and Photocatalytic Potentials

**DOI:** 10.3390/molecules27165184

**Published:** 2022-08-15

**Authors:** Sidra Batool Malik, Javed Iqbal Saggu, Asma Gul, Banzeer Ahsan Abbasi, Javed Iqbal, Saboora Waris, Yousef A. Bin Jardan, Wadie Chalgham

**Affiliations:** 1 Department of Biological Sciences, International Islamic University, Islamabad 44000, Pakistan; 2 Department of Physics, Quaid-i-Azam University, Islamabad 45320, Pakistan; 3 Department of Botany, Bacha Khan University, Charsadda 24420, Pakistan; 4 Department of Molecular Biology and Biochemistry, Shaheed Zulfiqar Ali Bhutto Medical University, Islamabad 44000, Pakistan; 5 Department of Pharmaceutics, College of Pharmacy, King Saud University, Riyadh 11451, Saudi Arabia; 6 Department of Mechanical and Aerospace Engineering, University of California, Los Angeles, CA 90095, USA

**Keywords:** silver nanoparticles, graphene, antibacterial, nanocomposites, degradation

## Abstract

Microbial pathogens and bulk amounts of industrial toxic wastes in water are an alarming situation to humans and a continuous threat to aquatic life. In this study, multifunctional silver and graphene nanocomposites (Ag)_1−x_(GNPs)_x_ [25% (x = 0.25), 50% (x = 0.50) and 75% (x = 0.75) of GNPs] were synthesized via ex situ approach. Further, the synthesized nanocomposites were explored for their physicochemical characteristics, such as vibrational modes (Raman spectroscopic analysis), optical properties (UV visible spectroscopic analysis), antibacterial and photocatalytic applications. We investigated the antimicrobial activity of silver and graphene nanocomposites (Ag-GNPs), and the results showed that Ag-GNPs nanocomposites exhibit remarkably improved antimicrobial activity (28.78% (*E. coli*), 31.34% (*S. aureus*) and 30.31% (*P. aeruginosa*) growth inhibition, which might be due to increase in surface area of silver nanoparticles (AgNPs)). Furthermore, we investigated the photocatalytic activity of silver (AgNPs) and graphene (GNPs) nanocomposites in varying ratios. Interestingly, the Ag-GNPs nanocomposites show improved photocatalytic activity (78.55% degradation) as compared to AgNPs (54.35%), which can be an effective candidate for removing the toxicity of dyes. Hence, it is emphatically concluded that Ag-GNPs hold very active behavior towards the decolorization of dyes and could be a potential candidate for the treatment of wastewater and possible pathogenic control over microbes. In the future, we also recommend different other in vitro biological and environmental applications of silver and graphene nanocomposites.

## 1. Introduction

Microbial pathogens causing disease are an alarming medical situation to human health and cause infections in various organs. To handle these pathogenic infections, certain steps should be required to manage an approach and to make some necessary strategies for these public health issues. In the recent era, the commonness of antibiotic-resistant bacteria and higher medical costs are threats to global health [1]. Microorganisms, including fungi and bacteria, are possible reasons for contamination and colonization process over the surface of the surgical apparatus. These highly contaminated apparatuses with various pathogens are a continuous threat to human health, thus following severe economic losses [2]. Therefore, it has become mandatory to synthesize and make possible utilization of new material to seize these health-related threats. In recent years, nanostructured materials have smoothed the way for designing innovative biocidal agents with improved and distinctive chemical and physical properties [3,4]. For this purpose, multiple nanoparticles such as silver (Ag) [5], zinc oxide (ZnO), titanium dioxide (TiO_2_), iron oxide (Fe_3_O_4_) [6], copper oxide (CuO) [7], magnesium oxide (MgO) [8] and nitric oxide (NO) nanoparticles [9] have been explored successfully and investigated the possible antibacterial properties. Thus, designing such nanoparticles with maximum inhibition over pathogenic agents could be a productive candidate as an antibacterial agent in the near future. It has been shown that nanoparticles (NPs) have the ability to make healthy interactions with biochemical moieties, which are the structural part of the microbial cells, leading to inhibition in the growth of microbes [10,11]. Carbon-natured nanostructures have been widely investigated due to their exclusive properties; among them, graphene-based nanomaterials (GBNs) have been in fame for the last decade due to their uniqueness with high surface-to-volume ratio, mechanically flexible and thermally stable [12,13]. Moreover, GBNs are in the limelight for their activity against bacterial growth, having toxicity towards both Gram-positive and Gram-negative bacteria. A study conducted by Yazhou et al. [14] has explored the antimicrobial activity of silver-decorated with graphene sheets against *E. coli*. The improved and excellent activity was observed due to the addition of graphene. The cytotoxicity has been successfully shown by two carbon-based nanomaterials, including graphene oxide (GO) and reduced graphene oxide (rGO) [14]. Metallic nanoparticles reflect uniqueness in their features which are found in direct relation to their morphological appearance, e.g., shape and size. Various metallic nanoparticles, particularly silver nanoparticles, have been researched as suitable candidates in medical applications, including diagnostics [15], drug delivery techniques [16], sanitization [17], wastewater treatment [18] and wound healing [19]. It has been found that silver nanoparticles (AgNPs) are unique when compared with other metallic nanoparticles (MNPs), having strong inhibition in microbial growth. Many researchers have investigated the release of Ag^+^ ions from AgNPs. When Ag^+^ ions are released, this results in the binding of Ag^+^ ions to the thiol group present in microbial enzymes, and their (Ag^+^–thiol)-binding interaction reacts with the microbial respiratory chain, creating oxidative stress and thus leading to cell death [20,21]. GO-Ag nanosheets depicted remarkable activity against *E. coli*, and inhibition in growth occurred due to cell deformation [22]. Another study showed the antibacterial activity of silver and graphene nanocomposites (designed through reduction method) against *Staphylococcus aureus* by agar well diffusion method. The inhibition in growth was observed due to the synergistic effect of Ag/graphene nanocomposites and the dispersibility of AgNPs on G structure [23]. GO-Ag and reduced GO-Ag nanostructures have an excellent inhibitory effect on microbes *E. coli* and *S. aureus* under identical conditions. Reduced graphene oxide showed much better than GO by providing more surface area to AgNPs [24]. It has been demonstrated during the study that maximum *E. coli* protein aggregation is seen by magnetic graphene (G-Fe_3_O_4_) and Tungsten oxide (G-WO_3_) nanocomposites than Fe_3_O_4_ and WO_3_ (without graphene) with low protein degradation alone [22,23]. Above mentioned findings suggest that G structure (graphene) is responsible for the deactivation of protein more than pure metals when they are being applied alone. Graphene having strong π–π stacking interactions has the ability to establish healthy contact with DNA in several groups present in pathogenic cells. For instance, the existence of graphene oxide (GO) together with Cu^2+^ can disturb DNA unwinding by chelating Cu^2+^ ions to oxygen atoms present on the GO nanosheets [24]. During the study, it was also reported that larger GO nanosheets with large surface areas display an important reduction in *E. coli* viability assay as compared to smaller nanosheets [25]. The biological issues in the environment are triggered by the massive utilization of dyes, organic wastes, insecticides and strong metallic compounds in aquatic environments (seas and rivers) on a worldwide scale. It has unlocked new visions for the beginning of vital and practical research on ecological protection [26]. About 10,000 different types of dyes and pigments are utilized by industries that result in the production of contaminants ~0.7 million per annum. Various studies have revealed that metallic nanocomposites designed on graphene-based nanosheets have shown remarkable results. A study conducted summed up the findings that CuO/rGO is three times more potent photocatalyst under visible light than CuO alone [27]. Nanocomposite Tungsten trioxide and reduced graphene oxide (WO_3_/rGO) nanocomposite have successfully ended in sonocatalytic degradation of Congo red (hazardous azo dye) from 56% to 94%; this happened due to the large surface area of WO_3_/rGO (62.03 m^2^/g) as compared to WO_3_ (44.79 m^2^/g) [28]. Titanium oxide and graphene oxide nanocomposites obtained via the hydrothermal method, TiO_2_ assisted with GO showed 57% degradation of salicylic acid [29]. Tin oxide (SnO_2_) deposition on graphene nanosheets has shown excellent gas-sensing performance electrochemically towards NO_2_ than other gases in comparison under the same conditions [30]. Industries are manufacturing highly toxic and non-biodegradable dyes, which are carcinogenic and affect human health and living organisms [31,32,33]. So far, various approaches have been employed in wastewater treatment, including thermal degradation, biodegradation and photodegradation [34]. Recently, nanomaterials have been shown to have great efficacy toward environmental pollution in various studies [35,36]. Degradation by photocatalysis is used in the degradation of colorants/organic pollutants [37,38,39,40,41,42]. Different metals and metallic oxides nanoparticles, e.g., Co-doped ZnO nanoparticles, have been successfully utilized under visible light in the degradation of organic toxic pollutants [43], Fe_2_O_3_ nanoparticles [35], TiO_2_ nanoparticles [44], polyoxometalate hybrid sustained on magnetic activated carbon, Au-FeS_2_ nanocomposites and MoS_2_/Ag nanocomposite [45,46]. Photocatalysis is one of the probable strategies utilized in degrading the toxicity level of organic dyes to CO_2_ and H_2_O and conversion to other harmless inorganic complexes without producing secondary pollutants. Various organic dyes, including bromophenol blue (BPB), methylene blue (MB) and methyl orange (MO), are normally used in a variety of fabric industries. Hence, they are responsible for causing severe biological hazards resulting in carcinogenic and mutagenic changes to the environmental and marine life [47,48]. In a previous study, AgNPs/GO modified with polyester fabric (AgNPs@GO/PET) showed active photocatalytic performance against 4-nitrophenol, methyl orange and methyl red; hence, GNPs have active potential in photodegradation of organic dyes [49]. Removal of methylene blue (cationic textile dye) has been successfully degraded by industrial IF slag steel up to 81.28% [50]. To cope with this issue, the degradation of toxic organic wastes (dyes) and depollution of wastewater is a key that can be accomplished by designing an effective catalytic agent that can promptly initiate the deactivation of organic waste pollutants [51,52]. Furthermore, the unique properties of graphene, such as its large surface area, good chemical inflexibility, marked electrical conductance and distinct absorptivity, design it into persistent material in many fields such as electronics, optics, photovoltaics, hydrogen storage, photocatalysis, and wastewater treatment [53]. Graphene is employed as a supportive material with other nanoparticles because of its larger surface area and potential utilization in wastewater treatment [54]. Herein, we investigated for the first time the incremental effects of graphene nanoparticles (GNPs) in varying ratios to AgNPs. For this purpose, (Ag)_1−x_(GNPs)_x_ nanocomposites with different weight ratios such as 25% (x = 0.25), 50% (x = 0.50) and 75% (x = 0.75) of GNPs were prepared via ex situ approach. The nanocomposites are thoroughly characterized for their optical, vibrational, crystalline, antibacterial and photocatalytic characteristics. This study involves the gradual increase in GNPs on AgNPs (silver nanoparticles) in different percentages; previously, various studies were conducted on graphene oxide and reduced graphene oxide, but here, we report on pure graphene combination with Ag and explore its activity through antibacterial assay and photocatalysis suggesting that Ag-GNPs nanocomposites showed strong bactericidal and photocatalytic activity as compared with AgNPs and GNPs alone. Interestingly, the strong antibacterial activity of Ag-GNPs is attributed to their large surface area. Furthermore, nanocomposites revealed active, visible light response and charge carrier separation efficiency, leading to improved photodegradation efficiency towards methylene blue (MB) dye. MB is used as a dye for photodegradation because of its vast utilization in the textile and printing industry. This dye is responsible for causing health abnormalities in humans and is also lethal to aquatic life [55]. Therefore, the prepared nanocomposites have great potential for antibacterial activity and for detoxification of organic dyes present in the industrial wastes.

## 2. Results

### 2.1. Optical Analysis

**Figure 1:**
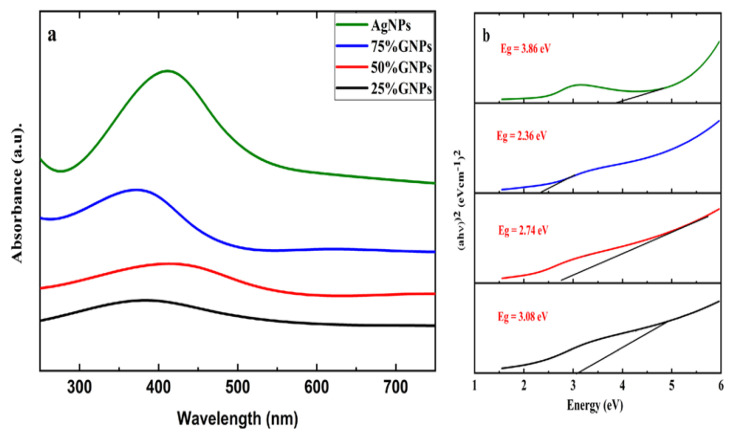
UV-Vis analysis of (**a**) AgNPs and GNP-Ag nanocomposites; (**b**) calculated energy bandgaps of AgNPs and Ag-GNPs nanocomposites.

Aqueous suspensions of AgNPs and (Ag)_1−x_(GNPs)_x_ were utilized to record the UV-visible spectra. The data were recorded in the 200–700 nm range through a spectrophotometer (Figure 1). The presence of AgNPs in the graphene nanoparticles was confirmed by the absorption peaks at 411 nm. This particular range of absorbance spectra shows the surface plasmon effect of AgNPs, confirming the attachment and presence of AgNPs on graphene nanostructure due to surface plasmon resonance (SPR) of silver nanoparticles, which is fairly good, as reported in earlier data [56]. It is observed that (Ag)_1−x_(GNPs)_x_ nanocomposites show slightly different spectra as compared to AgNPs. It might be due to the insertion of GNPs into AgNPs. The bond energy between C–C atoms (π–π*) present in GNPs is remarkably transformed after the addition of AgNPs; the reason for this phenomenon is due to the strong conjugated π-bond system due to the π–π stacking interaction between GNPs and AgNPs [57,58].

The Eg (energy band gap) of AgNPs and (Ag)_1−x_(GNPs)_x_ nanocomposites is calculated by using Tauc’s relation given below

(αhυ) n = A (hυ − Eg)

where A is a constant, hυ is photon energy, Eg = hc/λ, λ is the wavelength, α is the absorption coefficient and n is an integer; its value is measured by direct/indirect bandgap. For direct bandgap transitions n = 2, Eg obtained for AgNPs is 3.86 eV, which is fairly good in comparison with previously observed values, i.e., 3.73 eV 60; a reduction in bandgap with the addition of GNPs is observed, hence shrinking the energy band gap to 2.36 eV, 2.95 eV and 3.08 eV for 75% GNPs-Ag nanocomposite, 50% GNPs-Ag nanocomposite and 25% GNPs-Ag nanocomposite, respectively (Figure 1).

### 2.2. Raman Spectroscopic Analysis

**Figure 2:**
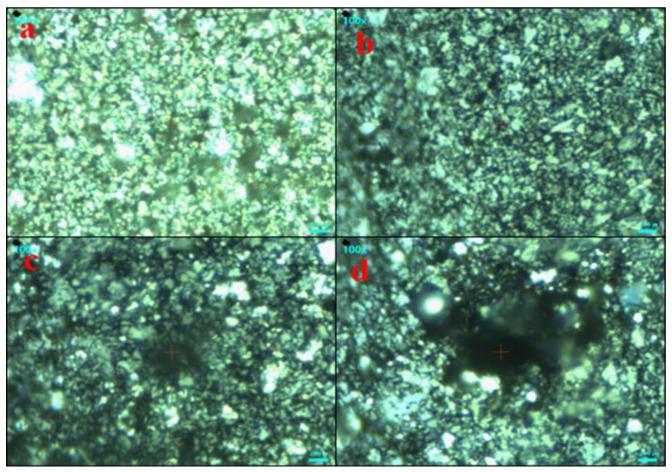
Raman images of AgNPs (**a**) and (Ag)_1−x_(GNPs)_x_; 25%, 50% and 75%GNPs (**b**–**d**).

**Figure 3:**
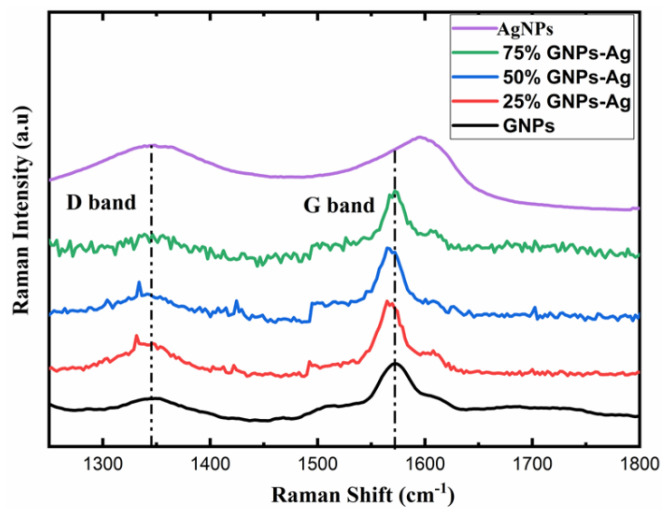
Raman analysis with active vibrational modes of AgNPs, GNPs and (Ag)_1-x_(GNPs)_x_.

For further investigation, AgNPs and (Ag)_1−x_(GNPs)_x_ nanocomposites vibrational modes were evaluated by exploiting the technique of Raman spectroscopy. It is an effective instrument for characterizing graphene-based nanocomposites. The images were obtained from Raman spectroscopy of AgNPs and Ag-GNPs with varying concentrations with a resolution of 100× (Figure 2). It is clear from the images that the increase in GNPs to AgNPs morphological changes appeared due to the intensification of GNPs. Raman analysis delivers valuable data based on the electronic and structural characterization of graphene. Raman spectra of pure AgNPs, graphene and (Ag)_1−x_(GNPs)_x_ indicate the peak difference observed between pure materials (AgNPs and graphene) and their nanocomposites occurred due to the attachment of AgNPs on graphene nanoplatelets (Figure 3). It was also observed that distinguished peaks of D and G bands were found at positions 1350 cm^−1^ and 1590 cm^−1^. Where the D band is associated with the breathing mode of the k-point, and the G band is associated with the tangential stretching mode of E_2_g phonon of sp2 carbon atoms [59]. Due to the origination of doubly degenerate phonon modes, G-band appears due to first-order Raman scattering in graphene (Figure 3). The D-band appears due to the second-order Raman scattering process [60]. The existence of variations between these two features leads to the formation of nanocomposites.

The appearance of the G-band is the key factor that specifies the formation of the synthesized nanocomposite; the peak shifting of AgNPs was observed from 1597 cm^−1^ up to 26 cm^−1^ with 75% GNPs, 31 cm^−1^ with 50% GNPs and 33 cm^−1^ with 25% GNPs with a new peak position at 1571 cm^−1^, 1566 cm^−1^ and 1564 cm^−1^, respectively. The shifting of peaks attributes their behavior due to the addition of GNPs, where the band is located at 1572 cm^−1^ in the graphene nanoparticles. This shifting in peak positions, as mentioned above, shows the transfer of electrical charge between the graphene nanostructures and AgNPs, hence ending up in the attachment of both nanostructures. The shifting of the D-band was also observed shift and appeared slightly broader. This shows that with the addition of GNPs in AgNPs, there are significant changes in peak positions and proves the successful formation of graphene nanocomposites [61,62].

### 2.3. Crystalline Structure Analysis (XRD)

**Figure 4:**
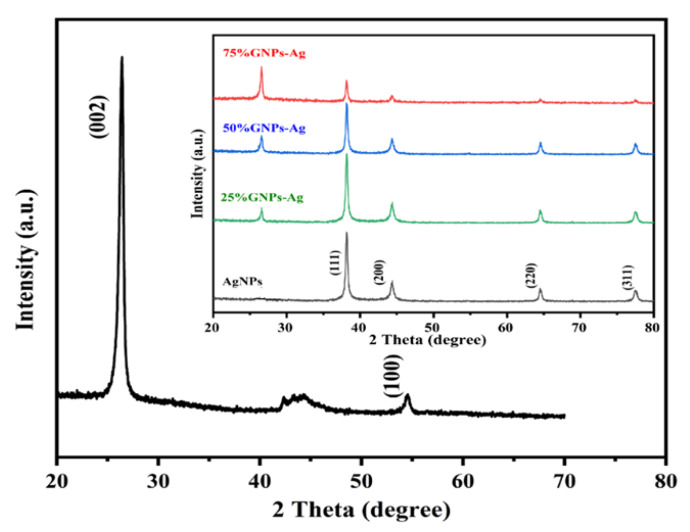
XRD spectral analysis of GNPs, AgNPs and Ag/G nanocomposites with varying ratios of GNPs (inset).

The XRD spectra of synthesized (Ag)_1−x_(GNPs)_x_ nanocomposites are shown in Figure 4. The XRD analysis confirmed the crystalline nature of AgNPs. The resulting Bragg peaks were in accordance with single and pure phase face-centered cubic AgNPs with JCPDS card no. 96-110-0137. The XRD spectra showed varying distinct major diffraction peaks with 2Ø values of 38.11, 44.30, 64.44 and 77.40, which corresponded to (111), (200), (220) and (311) Bragg’s reflections, respectively. The XRD spectrum obtained for GNPs showed the major diffraction peaks at 2Ø 26.54 (002) and 54.66 (100), confirming hexagonal structure with JCPDS card no 96-901-1578. The diffraction peaks observed in (Ag)_1−x_(GNPs)_x_ nanocomposites (Figure 1 insets) are at 2Ø 26.54 (002), 38.11 (111), 44.30 (200), 64.44 (220) and 77.40 (311). The peak at 2Ø 26.54 (002) in Ag/GNPs nanocomposite became intense as the % age of GNPs increased, hence confirming the anchoring of GNPs on to AgNPs surface. No additional XRD peaks were observed, indicating the successful synthesis of nanocomposites. The average size of AgNPs is calculated as ~24 nm, GNPs as ~32 nm and for (Ag)_1−x_(GNPs)_x_ nanocomposites was 34 nm. Our XRD configuration for described nanocomposites is consistent with earlier studies [63,64,65].

### 2.4. Morphological Analysis (SEM) 

**Figure 5:**
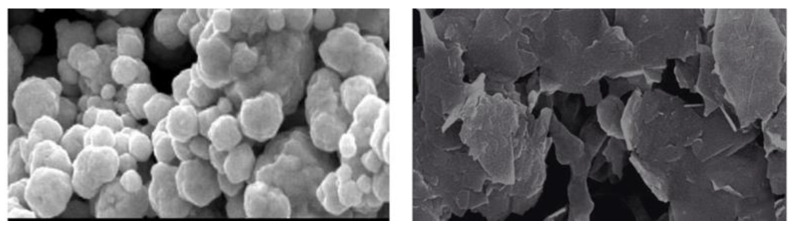
SEM analysis of AgNPs and GNPs.

**Figure 6:**
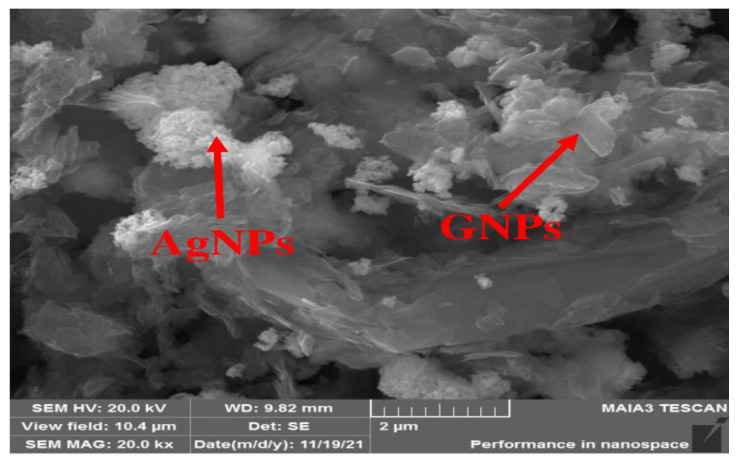
Morphology of 50% GNPs-Ag nanocomposites.

Morphological analysis was performed using scanning electron microscopy (SEM). Silver and graphene particles are visible with fine marginal ends, GNPs appearing as flat leafy shapes suggesting a platelet-like structure with fine margins having a well-composed shape (Figure 5). Images were obtained for silver and graphene nanocomposites (50% GNPs–Ag) (Figure 6). (Ag)_1−x_(GNPs)_x_ nanocomposites reflect unique features as graphene nanoparticles holding silver nanoparticles on their surface, confirming the successful synthesis of nanocomposites. The average nanocomposite size was calculated as almost equal to 29–34 nm.

### 2.5. Antibacterial Activity

**Figure 7:**
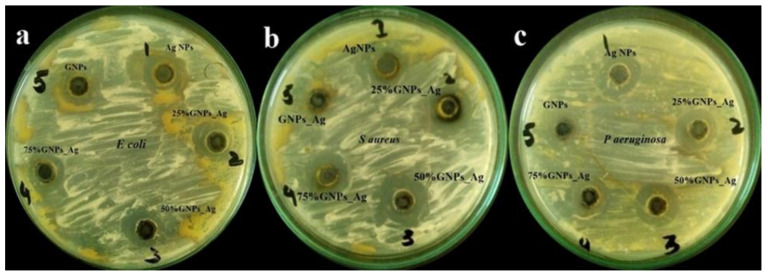
Zone of inhibition (ZOI) generated by (Ag)_1−x_(GNPs)_x_ in (**a**) *E. coli,* (**b**) *S. aureus* and (**c**) *P. aeruginosa*.

**Figure 8:**
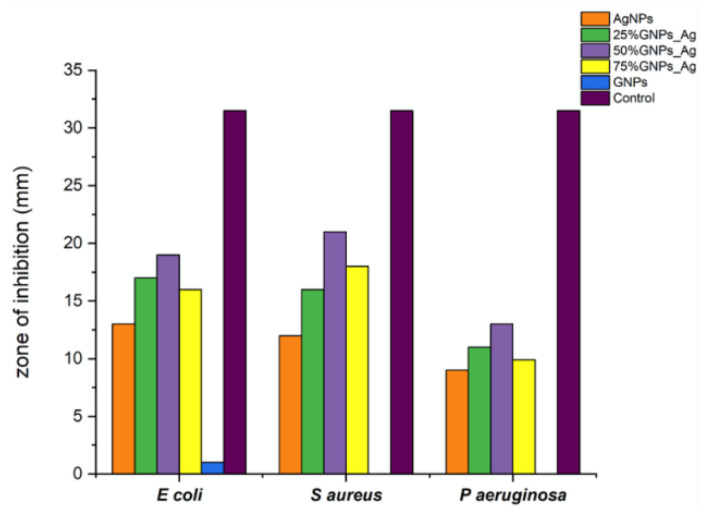
Zone diameters generated by AgNPs, (Ag)_1−x_(GNPs)_x_ and GNPs against *Escherichia coli, Styphylococcus aureus* and *Pseudomonas aeruginosa*.

The prepared nanocomposites, i.e., (Ag)_1−x_(GNPs)_x_, were investigated for their antibacterial performance against infectious bacterial strains, i.e., *Escherichia coli* (Gram-negative), *Staphylococcus aureus* (Gram-positive) and *Pseudomonas aeruginosa* (Gram-negative), and nanocomposite efficacy towards stains was evaluated (Figure 7 and Figure 8).

**Figure 9:**
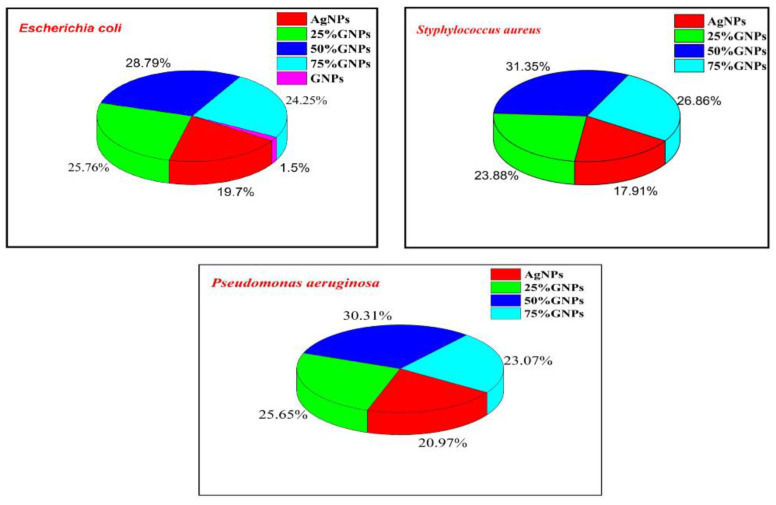
% Growth inhibition in bacterial pathogens with varying Ag-GNPs nanocomposites.

It was revealed from the results that the 50% GNP-AgNPs nanocomposites showed a maximum zone of inhibition against *S. aureus* with a zone diameter of 21 mm (Figure 9), while less inhibition was observed in *E. coli* and *P. aeruginosa* with the same ratio of nanocomposites (50% GNP-AgNPs) having inhibition zones of a diameter of about 13 mm and 9 mm, respectively. The AgNPs also showed antibacterial activity with maximum zone inhibition of 13 mm in *E. coli* as compared to *S. aureus* and *P. aeruginosa* with 12 mm and 9 mm of inhibition zone, respectively (Figure 7 and Figure 8). Hence, it was observed that the addition of GNPs with AgNPs enhanced the antibacterial activity. The antibacterial values are reported in Table 1.

The percentage growth inhibition was evaluated with the addition of GNPs to AgNPs; there was an enhancement in the antibacterial activity of nanocomposites (Figure 6). AgNPs alone showed less inhibition in all three bacterial strains as compared to Ag-GNPs nanocomposites. Among all nanocomposites, 50% GNPs-Ag are more towards inhibiting pathogens, i.e., 28.78% (*E. coli*), 31.34% (*S. aureus*) and 30.31% (*P. aeruginosa*) as compared to AgNPs, i.e., 19.78% (*E. coli*), 17.91% (*S. aureus*) and 20.97% (*P. aeruginosa*), respectively.

**Figure 10:**
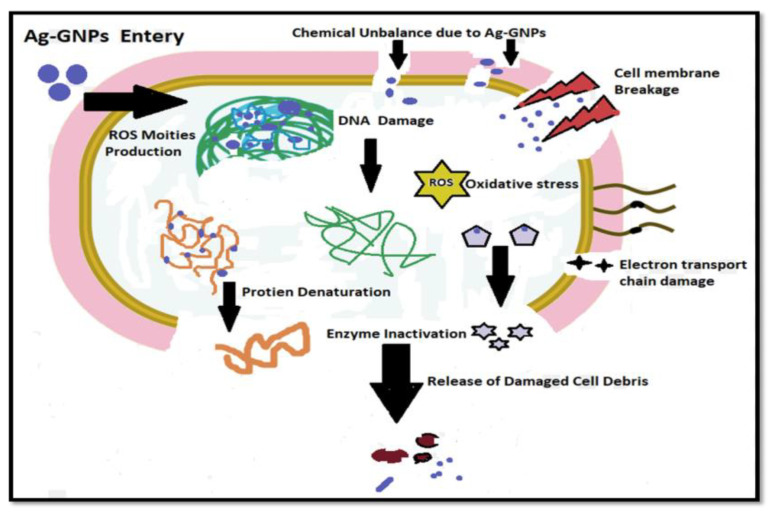
Proposed mechanism of bacterial cell death induced by Ag-GNPs nanocomposites.

The chemistry behind the mechanism of the antibacterial activity of AgNPs initiates with the discharge of Ag^+^ ions by silver nanoparticles (Figure 10). The silver ions thus make strong interaction with the sulfhydryl thiol groups, which are part of the proteins present on the bacterial cell membrane. The silver ions make a good attempt to replace the hydrogen cation (H+) from the sulfhydryl-thiol (-SH) group to create a much more stable S–Ag bond on the surface of a bacterial cell membrane, resulting in denaturation of the enzyme and causing a reduction in membrane permeability. Meanwhile, silver ions are expected to enter the cell and make alterations in DNA structure and cause cell death, while graphene enhances the surface area, with Ag^+^ having a synergistic effect against microbe’s toxicity [66].

### 2.6. Photocatalytic Activity

**Figure 11:**
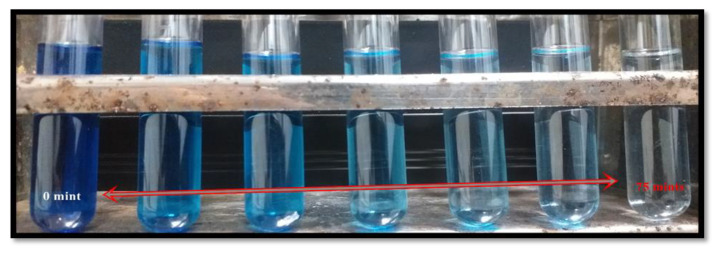
Dye decolorization within 75 min after exposure to visible light.

**Figure 12:**
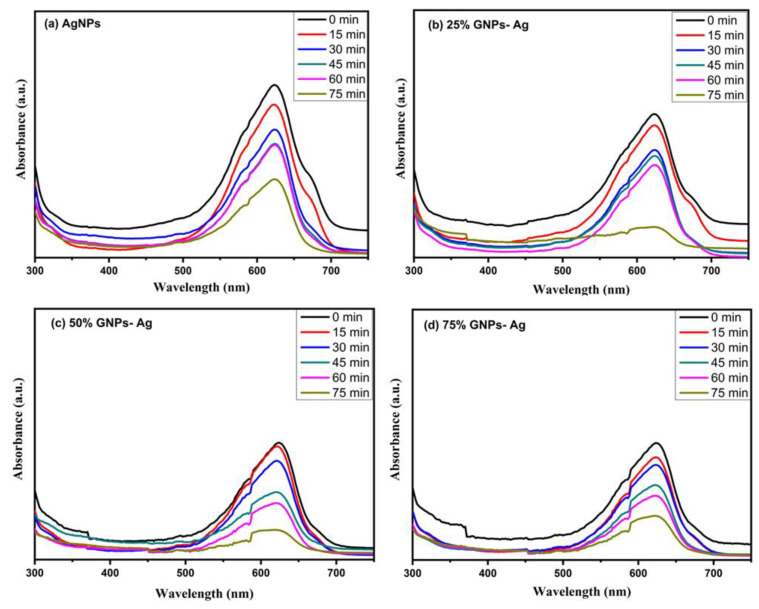
Photocatalytic degradation of MB under visible light irradiation at different time intervals with (**a**) AgNPs, (**b**) 25% GNPs-Ag nanocomposite, (**c**) 50% GNPs-Ag nanocomposite and (**d**) 75% GNPs-Ag nanocomposite.

The photocatalysis of methylene blue (MB) was recorded under visible light by utilizing AgNPs, GNPs and (Ag)_1−x_(GNPs)_x_ as nanocatalysts that dye degraded fully in 75 min after irradiating to visible light (Figure 11). Degradation of MB under visible light irradiation was recorded by the relative intensity of the UV-Vis spectra at the highest absorption peak at 667 nm (Figure 12). 

**Figure 13:**
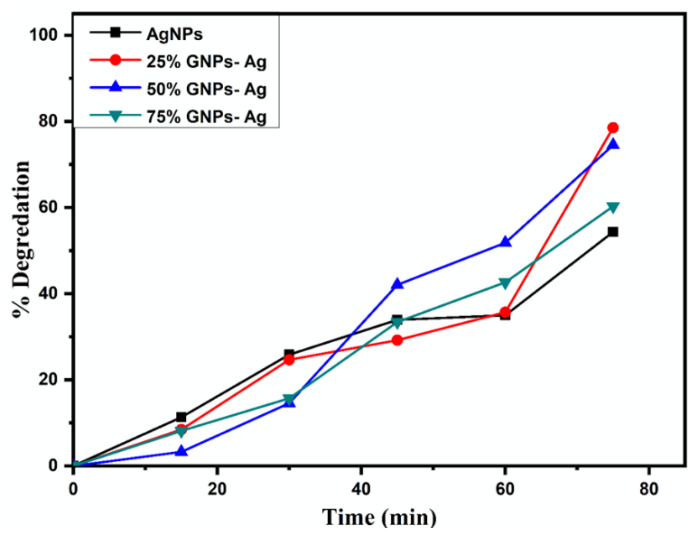
% Degradation of MB under visible light irradiation with (Ag)_1 − x_(GNPs)_x_.

The spectral analysis showed that the degradation of dye increased with an increase in irradiation time. The results presented that the (Ag)_1−x_(GNPs)_x_ degraded about 78.55% of the dye solution, while AgNPs degraded 54.34% of the dye (Figure 13). The % degradation was calculated with the given equation, and results were recorded.

$$\%\;\mathrm{Degradation}\;\mathrm{efficiency}\;=\frac{1-\;\mathrm{Co}}{\mathrm{Ct}}\times 100$$

Here, Ct is referred to as concentration at various intervals of time, Co means the initial concentration of aqueous suspension of MB, and Co and Ct are evaluated by means of Beer Lambert’s law. The absorption of MB depicts a reducing trend with the rise in exposure time in the light. Pseudo-first-order kinetics is applied for the photocatalytic activity of AgNPs and Ag/Graphene nanocomposites explicitly utilizing the expression below.

ln(Co/C) = kt

**Figure 14:**
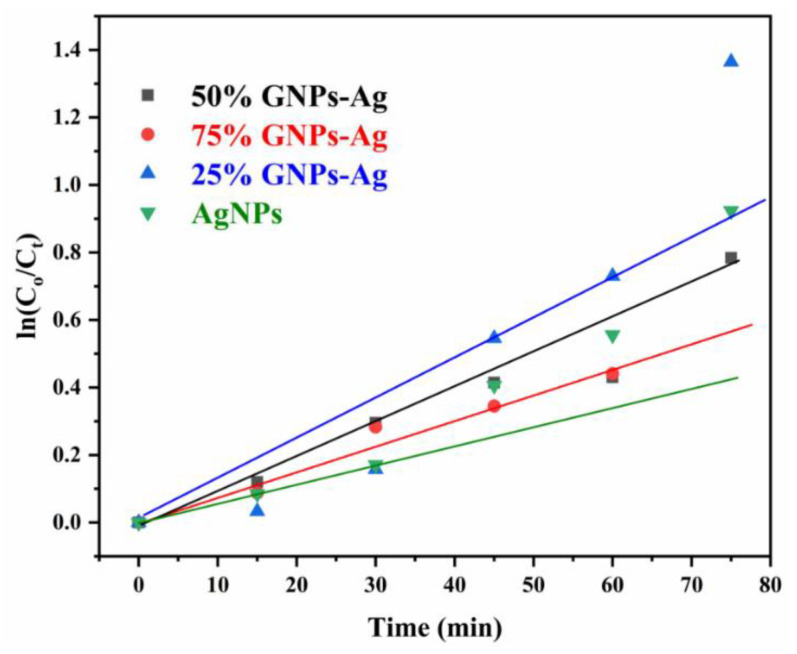
Pseudo-first-order kinetics of catalyst (Ag)_1−x_(GNPs)_x_ against MB.

The rate constants recorded by linear fitting the data are 0.7358 min^−1^, 0.9202 min^−1^, 0.9063 min^−1^ and 0.8873 min^−1^ for silver NPs, 25% GNPs-Ag, 50% GNPs-Ag and 75% GNPs-Ag nanocomposites, respectively (Figure 14). The constant rate variations are associated with the addition of graphene in the silver.

The photodegradation process is triggered by illuminating semiconductor photocatalyst under visible light irradiation. Positively charged holes (h^+^) in the valence band created by electrons present in the valence band excite the conduction band when high-energy photons get absorbed by them. In conduction band superoxide (·O^2^) anion radicals, which are strong oxidizing agents, are produced due to the interaction of electrons with O^2^ electron–acceptor moieties. Hydroxyl ion radical (·OH) takes place when water molecules make a reaction with a positively charged hole. Both these radicals become the reason for the degradation of toxic/contaminated organic dyes [67].

Photo nanocatalyst + hv (photon) → e-CB + h^+^VB

Photo nanocatalyst (e-) + O^2^ → (⋅O^2−^)

Photo nanocatalyst (h^+^) + H_2_O → ⋅OH + H^+^

Dye + ⋅OH → Degraded products

Dye + ⋅O^2^ → Degraded products

**Figure 15:**
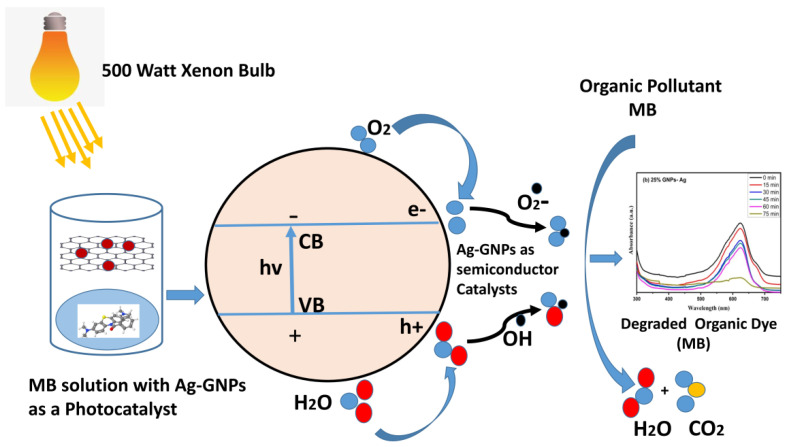
Schematic illustration of MB with (Ag)_1−x_(GNPs)_x_ nanocomposites.

The positive hole produced is responsible for making high oxidative potential organic pollutant (dye) molecules into direct oxidization to degradable products (Figure 15).

## 3. Discussion

Microbial pathogens causing disease are an alarming medical situation to human health and cause infections in various organs. To handle these pathogenic infections, certain steps should be required to manage an approach and to make some necessary strategies to tackle these public health issues. Metals with intrinsic antimicrobial characteristics such as silver, copper and zinc at their nanoscale comprise a special division of antimicrobials having broad-spectrum antimicrobial activity and are least toxic to humans. At the same time, these nanoparticles can be an active candidate as nanocatalysts in removing poisonous organic pollutants, which are discharged from industries as wastes. Silver nanoparticles gain much attention in biological applications related to health and infectious diseases. At the same time, toxicity caused by AgNPs has been reported by many researchers to overcome this issue; graphene is attached, as it has a large surface area with improved results as compared to pure metallic NPs. The present study has shown a simple way to synthesize silver-graphene nanocomposites via the ex situ method and evaluated their antibacterial and photocatalytic activity. For the confirmation of nanocomposites synthesis, they are characterized through UV-Visible analysis for optical and band gap calculation, while Raman analysis was conducted for vibrational modes. The energy difference present between the valence band and conduction band is responsible for the spectroscopic and chemical behaviors of the material, which is also involved in the conjugation system of the material from where various chemical reactions are evolved. LUMO is referred to as the lowest unoccupied molecular orbital referred to as π acceptor known as conduction band with the first unfilled energy level, in which the electron is kept moving freely in the lattice system, while HOMO is the uppermost (highest) occupied molecular orbital referred to as π donor known as valence band with last filled energy level for the molecules [68]. The reason behind the reduction in energy band gap essentially arises due to the creation of strong chemical interaction between GNPs and AgNPs in the synthesized nanocomposites. The healthy photocatalytic activity and decrease in absorption spectra of MB with time can be in good comparison with the shortened optical energy band gap. Fast photoexcitation of electrons is possible due to low band gaps. This would mark the visible light excited catalyst to photodegrade the adsorbed dye speedily. The small band gap of GNPs-Ag nanocomposites has specified its possible utilization as an operative photocatalyst in a visible light situation [69,70]. Raman analysis showed that the sharpness of both bands (D and G bands) was boosted dramatically after fixing of AgNPs on the surface of graphene nanostructures; hence it is clear proof of the successful attachment of AgNPs to the graphene. This sharpness in bands confirms the intercalation/decoration of AgNPs on graphene nanosheets, thus revealing the combination of graphene nanosheets with the Ag–graphene nanocomposite [71]. It has been reported in previous studies that silver has some cytotoxic effects while used in biological systems; hence, to overcome or mask this issue, it is combined with graphene to show better activity with low toxic effects. (Ag)_1−x_(GNPs)_x_ nanocomposites were applied on three bacterial strains; among these nanocomposites, 50% GNPs-Ag showed maximum inhibition in bacterial growth; the bacterial cell death might be due to oxidative stress caused by nanocomposites that consequently leads to DNA fragmentation [72,73]. It has been reported in previous studies that Ag^+^ had a role in the antibacterial activity by decreasing inhibition zones that were larger than silver nanoparticles. However, Ag^+^ ions have disadvantages as well, as they are heavy and toxic metals that have adverse effects on humans [74]. The comparative analysis for photodegradation of these nanocomposites with previous studies confirmed their ability as an active nanocatalyst reported in Table 2. Hence, silver nanoparticles (AgNPs) combined with GNPs to reduce the toxic properties of Ag^+^ so that they are deemed safe for human health [75].

## 4. Materials and Methods

### 4.1. Chemicals

Prepared silver nanoparticles (99.99%, Guangzhou Hongwu Material Technology, Guangzhou, China), graphene nanoparticles (100%, Knano, Xiamen, China), de ionized water, methylene blue and nutrient agar media (99.98%, Sigma Aldrich, Saint Louis, MO, USA) were used as chemical materials in this study.

### 4.2. Synthesis of (Ag)_1−x_(GNPs)_x_ Nanocomposites

(Ag)_1−x_(GNPs)_x_ nanocomposites with different weight ratios such as 25% (x = 0.25), 50% (x = 0.50) and 75% (x = 0.75) of GNPs have been synthesized via ex situ approach. A sample with 25% GNPs was prepared by a simple method: 25% of GNPs and 75% of AgNPs were dispersed in deionized water and ultra-sonicated for 30 min until a fruitful homogenous solution was achieved. Further, the Ag/GNPs aqueous solution was stirred at 500 rpm for half an hour at room temperature. The obtained nanocomposites were dried at 80 °C in an oven for 16 h, followed by crushing in mortar and pestle and grounding into fine powder. Nanocomposites with 50% and 75% GNPs were synthesized by adopting using the same method.

### 4.3. General Characterizations

The band gap and optical studies of prepared nanocomposites were evaluated by spectrophotometer. The absorbance spectra were obtained by Evolution 300 UV-Vis spectrophotometer. Vibrational properties of nanocomposites were observed at room temperature; Raman spectra were taken by NOST Raman spectroscopy (wavelength of laser is 532 nm). Morphological studies were investigated by scanning electron microscopy. VEGA3 TESCAN instrument was used for morphological analysis. XRD technique was utilized to examine the crystalline phase of prepared samples. XRD of the synthesized samples was carried out with the help of a PANalytical Empyrean diffractometer. The prepared nanostructured size was calculated by following the Scherrer formula:

D = 0:9 ʎ = β cos θ(1)

### 4.4. Antibacterial Assay

Three bacterial strains, *Escherichia coli* (ATCC: 15224), *Styphylococcus aureus* (ATCC: 25923), and *Pseudomonas aeruginosa* (ATCC: 9721), were collected from the Department of Pharmacy, Quaid-i-Azam University, Islamabad, Pakistan and antibacterial activity of prepared (Ag)_1−x_(GNPs)_x_ nanocomposites was performed using agar well diffusion method. In this method, the AgNPs and (Ag)_1−x_(GNPs)_x_ nanocomposites dilutions (1 mg/1 mL) were made in deionized water. Then, solutions were sonicated for about 30 min. Healthy bacterial colonies were attained in nutrient broth at 37 °C. With the help of a sterile cotton swab nutrient agar plates were inoculated with bacterial colonies. Then, 6 mm diameter holes (wells) were punctured aseptically on the agar surface via a sterile tip. These wells were filled by picking 10 μL of each AgNPs and (Ag)_1−x_(GNPs)_x_ nanocomposites with the help of a micropipette. These inoculated plates with antibacterial agents were incubated at 4 °C for 2–3 h to allow uniform diffusion of samples into agar medium, followed by incubation at 37 °C for 12–24 h. These assays were performed in triplicate, and inhibiting zones were calculated in millimeters [81,82].

### 4.5. Measuring of Photocatalytic Properties

The photocatalysis of methylene blue under visible light was conducted to investigate the photocatalytic effect of AgNPs and (Ag)_1−x_(GNPs)_x_ nanocomposites. UV-Vis spectrophotometer was utilized to record the absorbance data. In total, 40 mg of catalyst was utilized with 100 mL of water as the initial volume to perform photocatalytic experiments. A 3 ppm aqueous suspension of methylene blue (MB) was used in all experiments. Before exposure to visible light, the dye suspension was magnetically stirred in the dark for 30 min for the possible achievement of adsorption/desorption equilibrium. After irradiation with visible light using 500 Watts xenon bulb fixed at 15 cm above the reaction mixture in a box to avoid any external interruption, the pH of the reaction mixture was set at 8. A 4 mL of solution was taken out at defined intervals (15 min) following centrifugation at 4000 rpm for 10 min to eliminate photo nanocatalyst. The absorbance data of all the samples were recorded by UV-Vis spectrophotometer.

## 5. Conclusions

In this study, novel (Ag)_1−x_(GNPs)_x_ nanocomposites were successfully synthesized via an ex situ approach. Optical and vibrational investigations of these nanocomposites revealed the anchoring of graphene nanoplatelets onto AgNPs. Herein, (Ag)_1−x_(GNPs)_x_ nanocomposites showed significant antibacterial activities against *E. coli*, *S. aureus* and *P. aeruginosa*. In total, 50% GNPs-Ag nanocomposites showed strong antibacterial activity (31.34%) with *S. aureus* compared to AgNPs, which was 17.91%. Visible light irradiation showed maximum photodegradation of methylene blue (78.55%) with 25% GNPs-Ag as compared to pure AgNPs, which was 54.35%. From the results, it is concluded that silver nanoparticles are more effective in combination with graphene. Ag-GNPs can be utilized as an effective multifunctional material in preventing the spread of pathogens, which can also serve as an active candidate for the detoxification of innumerable toxic organic compounds present in industrial wastes. In the future, we recommend other different in vitro and in vivo biological and environmental applications of silver and graphene nanocomposites.

## Figures and Tables

**Table 1:** ZOI generated by AgNPs, (Ag)_1−x_(GNPs)_x_ and GNPs against bacterial strains.

Nanosystems (1 mg/mL)	Bacterial Stains (ZOI in mm)		
	*Escherichia coli*	*Styphylococcus aureus*	*Pseudomonas aeruginosa*
Control	32	31.5	31.5
AgNPs	13	12	09
25%GNPs-Ag	17	16	11
50%GNPs-Ag	19	21	13
75%GNPs-Ag	16	18	9.9
GNPs	01	0	0

**Table 2:** Comparative analysis between photocatalytic degradation of different Ag–graphene-based nanostructures, synthesized by various methods against dye.

Material	Synthesis Method	Size	Morphology	Dye	Light Source	Exposure Time	% Degradation	Rate m^−1^	Ref
Ag@rGO	Hydrothermalapproach	28.3 nm	Lamellar and sheet-like	MB	Mercury lamp (400 W)	20 min	69	0.7459	[76]
TiO_2_-AgFe-rGO	Sole gel method	25 nm	Rough spherical	MO	UV irradiation	30 min	71.5	-	[77]
Ag decorated rGO	Hummers method	90 nm	Layered sheet-like	MB	sunlight	180 min	72	1.3 × 10^−2^	[78]
RGO/PEI/Ag	In situ co-reduction	60 nm	Sheet on sheet like	MB	Mercury lamp (100 W)	120 min	-	0.95487	[79]
Ag/ZnO/g-C_3_N_4_	Green Synthesis	44 nm	Spherical on sheet	MB	UV Light	120 min	78.40	-	[80]
(Ag)_1-x_(GNPs)_x_	Ex-situ	34 nm	Spherical AgNPs on GNP platelets	MB	Vis Light Xenon lamp (500 W)	60 min	78.55	0.92028	Current study

## Data Availability

Data are part of the article.

## Sample Availability

Samples of the compounds are available from the authors.
